# The integrative clinical impact of tumor-infiltrating T lymphocytes and NK cells in relation to B lymphocyte and plasma cell density in esophageal and gastric adenocarcinoma

**DOI:** 10.18632/oncotarget.19437

**Published:** 2017-07-21

**Authors:** Maria C. Svensson, Carl Fredrik Warfvinge, Richard Fristedt, Charlotta Hedner, David Borg, Jakob Eberhard, Patrick Micke, Björn Nodin, Karin Leandersson, Karin Jirström

**Affiliations:** ^1^ Department of Clinical Sciences Lund, Oncology and Pathology, Lund University, Lund, Sweden; ^2^ Department of Immunology, Genetics and Pathology, Uppsala University, Rudbeck Laboratory, Uppsala, Sweden; ^3^ Cancer Immunology, Department of Translational Medicine, Lund University, Malmö, Sweden

**Keywords:** T lymphocytes, B lymphocytes, esophageal cancer, gastric cancer, prognosis

## Abstract

**Background:**

Several studies have demonstrated a prognostic impact of tumor-infiltrating T lymphocytes and natural killer (NK) cells in esophageal and gastric adenocarcinoma, but whether these associations differ by the density of tumor-infiltrating immune cells of the B cell lineage remains largely unknown.

**Results:**

High infiltration of any T and NK lymphocytes investigated was in general associated with a favorable prognosis, but the strongest beneficial prognostic impact was seen in combination with high B lymphocyte infiltration. These findings were most evident in gastric cancer, where significant interactions in relation to OS were observed for CD3^+^, CD8^+^ and FoxP3^+^ with CD20^+^ cells (p_interaction_ =0.012, 0.009 and 0.007, respectively) and for FoxP3^+^ with IGKC^+^ cells (p_interaction_ =0.034). In esophageal tumors, there was only a significant interaction for CD3^+^ and CD20 ^+^ cells (p_interaction_ =0.028).

**Methods:**

Immunohistochemistry and automated image analysis was applied to assess the density of T lymphocytes (CD3^+^, CD8^+^, FoxP3^+^) and NK cells (NKp46^+^) in chemoradiotherapy-naïve tumors from a consecutive cohort of 174 patients with resected esophageal or gastric adenocarcinoma. The density of B lymphocytes (CD20^+^) and plasma cells (IGKC^+^) had been assessed previously. Kaplan-Meier analysis and Cox proportional hazard's modelling was applied to examine the impact of the investigated markers on time to recurrence (TTR) and overall survival (OS).

**Conclusions:**

These data support that the antitumoral effects of tumor-infiltrating T lymphocytes in esophageal and gastric adenocarcinoma may be largely dependent on a functional interplay between T and B lymphocytes or plasma cells.

## INTRODUCTION

Esophageal and gastric cancers represent a substantial proportion of cancer cases and deaths worldwide [1]. The prognosis is poor, especially in Western populations, with 5-year survival rates less than 40%. Addition of neoadjuvant and/or adjuvant chemotherapy or chemoradiotherapy has been shown to improve survival in patients with resectable gastric and esophageal cancer [2–7]. However, in order to further decrease the high mortality associated with these diseases, there is an urgent need to take further steps towards improved treatment stratification by the identification and clinical implementation of prognostic and response predictive biomarkers.

Several studies have demonstrated a prognostic impact of tumor-infiltrating lymphocytes (TILs) in a multitude of cancers [8, 9] including esophageal and gastric adenocarcinoma. In gastric cancer, dense infiltration of CD3^+^ and CD8^+^ TILs has been associated with an improved prognosis [10]. The prognostic value of FoxP3^+^ regulatory T cells (Tregs) appears to be more ambiguous, with some studies demonstrating an association with an improved prognosis and some with a poor prognosis [11–13]. In esophageal adenocarcinoma, no convincing prognostic value has been demonstrated for CD3^+^ or CD8^+^ T cells and high density of FoxP3^+^ Tregs has been associated with a poor prognosis [14, 15].

Another cell of interest is the tumouricidal natural killer (NK) cell [16]. A favorable prognostic value of CD56^+^ NK/NKT cell infiltration has been demonstrated in many tumor types such as non-small lung cancer carcinoma [17], colorectal carcinoma [18] and periampullary adenocarcinoma [19]. To our knowledge no studies evaluating the prognostic value of NK cells in gastric or esophageal cancer have yet been published.

While most studies relate to the prognostic significance of tumor-infiltrating T lymphocytes and NK cells, less attention has been paid to the influence of B cells. Of note, B cells make up a significant component of the lymphocytic infiltrate in several types of solid tumors [20] and dense infiltration of B cells has been demonstrated to correlate with an improved outcome in e.g. breast, ovarian, colorectal and gastro-esophageal cancer [21–24]. Moreover, a synergistic prognostic effect has been demonstrated for dense infiltration of both CD8^+^ T cells and CD20^+^ B cells in high-grade epithelial ovarian cancer [25]. To our best knowledge, no studies have yet reported whether the prognostic impact of T lymphocytes and NK cells differs according to the density of B cells and plasma cells in esophageal or gastric cancer.

Therefore, the aim of this study was to examine the prognostic impact of various subsets of tumor-infiltrating T cells and NK cells in relation to clinical outcome, alone and stratified according to B cell and plasma cell density, in esophageal and gastric adenocarcinoma. To this end, we examined tumors from a consecutive, retrospective cohort of patients with chemoradiotherapy-naïve resected esophageal or gastric adenocarcinoma.

## RESULTS

### Distribution and intercorrelation of different lymphocyte subsets and their associations with clinicopathological factors

Immunohistochemistry and automated image analysis was applied to assess the density of T lymphocytes (CD3^+^, CD8^+^, FoxP3^+^) and NK cells (NKp46^+^) in tumors from a previously described consecutive cohort of 174 patients with resected esophageal or gastric adenocarcinoma, none of whom had been subjected to neoadjuvant chemoradiotherapy or perioperative chemotherapy [26–32].

**Figure 1:**
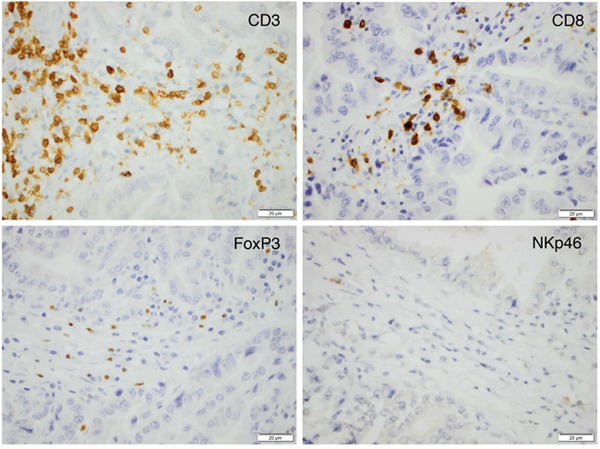
Sample immunohistochemical images (magnification x 40) of the investigated immune cell subsets in an esophageal T2N0M0 adenocarcinoma

The density of B lymphocytes (CD20^+^) and plasma cells (IGKC^+^) had been assessed previously [33]. CD3 expression could be evaluated in 172 (98.9%) cases, CD8 expression in 171 (98.3%) cases, FoxP3 expression in 173 (99.4%) cases and NKp46 expression in 165 (94.8%) cases. Sample IHC images of the investigated immune cell subsets are shown in Figure 1.

The intercorrelations between different lymphocyte subsets, in the entire cohort and stratified by location, are shown in Table 1. In the entire cohort, there was a strong to very strong correlation between CD3^+^, CD8^+^ and FoxP3^+^ cells, with similar findings in esophageal and gastric cancer, respectively. The intercorrelations between NKp46^+^ cells and the different T lymphocyte subsets were allover moderate. In the entire cohort, CD20^+^ B cells correlated strongly, and IGKC^+^ plasma cells correlated moderately to strongly, with CD3^+^, CD8^+^ and FoxP3^+^ cells respectively, with similar findings in esophageal and gastric cancer for CD20^+^ cells and stronger correlations for IGKC^+^ plasma cells in gastric cancer. The intercorrelations between NKp46^+^ cells and CD20^+^ cells were allover moderate, whereas the intercorrelations between NKp46^+^ cells and IGKC^+^ plasma cells were allover strong.

**Table 1:** Interrelationship between investigated lymphocyte subsets in the entire cohort, esophageal and gastric cancer

	Entire cohort				Esophagus				Stomach			
	CD3^+^	CD8^+^	FoxP3^+^	NKp46^+^	CD3^+^	CD8^+^	FoxP3^+^	NKp46^+^	CD3^+^	CD8^+^	FoxP3^+^	NKp46^+^
**CD3^+^**												
*R*		0.877	0.718	0.307		0.855	0.707	0.312		0.876	0.701	0.280
*p*		<0.001	<0.001	<0.001		<0.001	<0.001	0.002		<0.001	<0.001	0.018
*n*		169	171	164		94	96	93		75	75	71
**CD8^+^**												
*R*	0.877		0.619	0.281	0.855		0.652	0.270	0.876		0.569	0.264
*p*	<0.001		<0.001	<0.001	<0.001		<0.001	0.009	<0.001		<0.001	0.026
*n*	169		171	163	94		96	92	75		75	71
**FoxP3^+^**												
*R*	0.718	0.619		0.302	0.707	0.652		0.280	0.701	0.569		0.327
*p*	<0.001	<0.001		<0.001	<0.001	<0.001		0.006	<0.001	<0.001		0.005
*n*	171	171		165	96	96		94	75	75		71
**NKp46^+^**												
*R*	0.307	0.281	0.302		0.312	0.270	0.280		0.280	0.264	0.327	
*P*	<0.001	<0.001	<0.001		0.002	0.009	0.006		0.018	0.026	0.005	
n	164	163	165		93	92	94		71	71	71	
**CD20^+^**												
*R*	0.607	0.531	0.574	0.303	0.548	0.458	0.553	0.303	0.599	0.549	0.568	0.277
*P*	<0.001	<0.001	<0.001	<0.001	<0.001	<0.001	<0.001	0.003	<0.001	<0.001	<0.001	0.020
n	168	168	170	161	94	94	96	92	74	74	74	70
**IGKC^+^**												
*R*	0.451	0.403	0.350	0.449	0.415	0.360	0.290	0.421	0.529	0.460	0.441	0.594
*P*	<0.001	<0.001	<0.001	<0.001	<0.001	<0.001	0.004	<0.001	<0.001	<0.001	<0.001	<0.001
n	171	171	173	165	96	96	98	94	75	75	75	71

R = Spearman's correlation coefficient, p = p-value, n = number of cases available for analysis.

The associations of the investigative lymphocyte subsets with clinicopathological parameters in the entire cohort are shown in Table 2. High density of CD3^+^ cells was significantly associated with lower N stage and lower grade, and the density of CD3^+^ cells was significantly higher in the stomach than in the esophagus. High density of CD8^+^ cells was significantly associated with higher age, lower N stage, lower grade and the density of CD8^+^ cells was significantly higher in the stomach than in the esophagus. High density of FoxP3^+^ cells was significantly associated with lower T stage, lower N stage, and R0 status. High density of NKp46^+^ cells was significantly associated with lower T stage.

**Table 2:** Associations of investigative lymphocyte subsets with clinicopathological parameters in the entire cohort

Factor	CD3^+^		CD8^+^		FoxP3^+^		NKp46^+^	
	mean, median(range)	*P(n)*	mean, median(range)	*P(n)*	mean, median(range)	*P(n)*	mean, median(range)	*P(n)*
**Age**		0.562		0.034		0.581		0.139
≤ average	535.41,514.25 (40.00-1954.00)	(86)	198.88,141.50 (1.00-1073.00)	(86)	125.41,110.00 (2.00-480.00)	(86)	1.89,0.50(0.00-19.50)	(83)
>average	590.09,518.75 (12.00-1709.00)	(86)	282.07,221.50 (3.50-1211.00)	(85)	138.23,92.50 (3.00-700.00)	(87)	1.93,0.75(0.00-15.00)	(82)
**Gender**		0.829		0.600		0.610		0.877
Female	561.91,514.51 (61.50-1389.50)	(39)	266.31,152.00 (11.50-903.50)	(39)	112.91,88.50 (2.00-325.00)	(39)	1.99,0.50(0.00-14.50)	(37)
Male	563.00,514.00 (12.00-1954.00)	(133)	232.53,172,25 (1.00-1211.00)	(132)	137.37,101.25 (3.00-700.00)	(134)	1.89,0.50(0.00-19.50)	(128)
**T-stage**		0.054		0.110		0.001		0.002
T1	464.60,442.00 (73.00-1017.50)	(19)	197.26,203.00 (3.50-481.50)	(17)	161.06,136.25 (17.50-472.50)	(18)	4.70,4.00(0.00-19.50)	(15)
T2	741.47,659.25 (12.00-1954.00)	(32)	318.64,223.50 (1.00-1130.00)	(32)	190.91,168.75 (16.00-700.00)	(32)	190.91,168.75 (16.00-700.00)	(32)
T3	558.32,512.25 (21.00-1767.00)	(94)	238.49,1512.00 (5.00-1211.00)	(95)	121.18,93.75 (3.00-480.00)	(96)	1.63,0.50(0.00-16.00)	(95)
T4	435.44,380.00 (57.70-1188.50)	(27)	180.50,127.50 (2.00-905.00)	(27)	80.39,62.50 (2.00-185.00)	(27)	0.76,0.50(0.00-6.00)	(25)
**N-stage**		0.004		0.004		0.002		0.188
N0	722.94,737.50 (12.00 −1954.00)	(58)	326.15,247.50(1.00 −1211.00)	(58)	178.25,157.50 (16.00 −635.00)	(58)	3.14,1.00(0.00 −19.50)	(53)
N1	521.05,481.00 (105.50-1447.50)	(30)	225.22,126.00(6.50-905.00)	(29)	114.87,93.25 (13.50-294.00)	(30)	1.47,0.00(0.00-80.50)	(30)
N2	429.43,346.00 (61.50-1031.00)	(41)	158.04,121.50(2.00-596.50)	(41)	94.95,67.00(2.00-340.00)	(41)	0.91,0.50(0.00 −5.50)	(41)
N3	502.91,450.50 (21.00 −1445.50)	(43)	212.85,143.50(3.50-1020.00)	(43)	116.68,76.50(3.00 −700.00)	(44)	1.66;0.00(0.00 −11.00)	(41)
**M-stage**		0.321		0.328		0.073		0.414
M0	576.37,524.50 (12.00-1954.00)	(154)	248.06,175.50 (1.00-1211.00)	(152)	135.77,107.50 (3.00-635.00)	(154)	2.01,0.50(0.00-19.50)	(148)
M1	321.17,261.50 (149.50-552.50)	(3)	96.33,52.50 (33.50-203.00)	(3)	72.17,60.00 (31.50-125.00)	(3)	0.17,0.00(0.00-0.50)	(3)
**Grade**		0.014		0.045		0.580		0.210
Low	613.73,572.75 (12.00-1767.00)	(112)	269.16,194.00 (1.00-1211.00)	(112)	131.84,95.50 (2.00-700.00)	(113)	2.13,0.50(0.00-16.00)	(108)
High	467.59,404.25 (40.00-1954.00)	(60)	185.33,145.00 (5.00-764.50)	(59)	131.89,98.75 (7.00-410.00)	(60)	1.50,0.50(0.00-19.50)	(57)
**Residual tumor status**		0.251		0.439		0.017		0.663
R0	594.96,525.06(67.00-1954.00)	(119)	250.00,176.00(1.00-1130.00)	(117)	148.22,116.25(11.00-700.00)	(118)	2.05,0.50(0.00-19.50)	(112)
R1	480.90,425.75(12.00-1658.00)	(44)	1.74,0.25(0.00-14.00)	(44)	97.12,75.00(2.00-385.00)	(46)	1.74,0.250.00-14.00	(44)
R2	537.11,577.00(149.50-803.50	(9)	236.06,253.50(33.50-388.00)	(9)	94.83,60.00(30.00-265.00)	(9)	1.06,0.50(0.00-6.00)	(9)
**Location**		0.046		0.047		0.437		0.064
Esophagus	518.63,423.00 (12.00-1954.00)	(97)	209.74,140.25 (2.00-1211.00)	(96)	126.91,90.50 (3.00-635.00)	(98)	1.33,0.50(0.00-19.50)	(94)
Stomach	619.81,578.50 (67.00-1767.00)	(75)	279.26,216.00 (1.00-1130.00)	(75)	138.32,115.00 (2.00-700.00)	(75)	2.69-0.50(0.00-16.00)	(71)

R0 = no residual tumor (free resection margins according to pathology report), R1 = possible microscopic residual tumor (narrow or compromised resection margins according to pathology report), R2 = macroscopic residual tumor (according to surgery report).

N1 = metastasis in 1–2 regional lymph nodes, N2 = metastasis in 3–6 regional lymph nodes, N3 = metastasis in 7 or more regional lymph nodes.

### Prognostic significance of T, NK/T and NK cells

**Figure 2:**
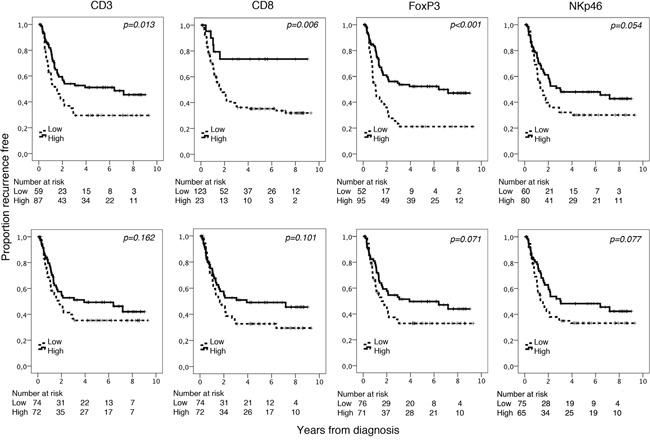
Kaplan-Meier analysis of time to recurrence in strata according to high and low total density of CD3^+^, CD8^+^, FoxP3^+^ and NKp46^+^ cells in th entire cohort, defined by CRT analysis (top row) and using the median value as cutoff (bottom row)

Kaplan-Meier analyses of the prognostic impact of the investigated lymphocyte subsets in relation to time to recurrence (TTR) in the entire cohort are shown in Figure 2. Using the classification and regression tree (CRT) based cutoff, high CD3^+^, CD8^+^ and FoxP3^+^ density was significantly associated with a prolonged TTR (p=0.013, p=0.006 and p<0.001, respectively). The association between high NKp46^+^ and TTR did not reach significance (p=0.054). Using the median value as cutoff, no significant associations with TTR were found.

**Figure 3:**
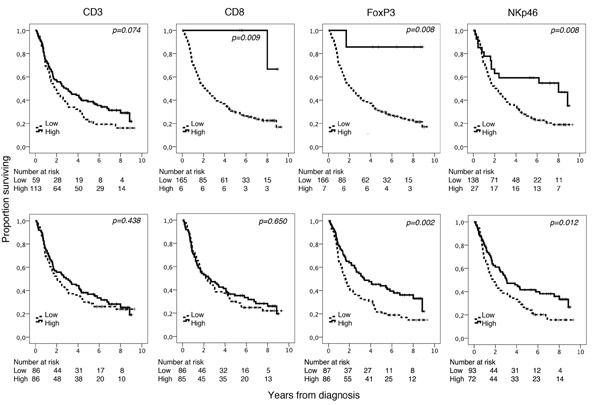
Kaplan-Meier analysis of overall survival in strata according to high and low total density of CD3^+^, CD8^+^, FoxP3^+^ and NKp46^+^ cells in the entire cohort, defined by CRT analysis (top row) and using the median value as cutoff (bottom row)

Kaplan-Meier analyses of the prognostic impact of the investigated lymphocyte subsets in relation to overall survival (OS) in the entire cohort are shown in Figure 3. Using CRT based cutoff, high density of CD8^+^, FoxP3^+^ and NKp46^+^ were all significantly associated with a prolonged OS (p=0.009, p=0.008 and p=0.008, respectively). Using the median value as cutoff high FoxP3^+^ and NKp46^+^ density was significantly associated with a prolonged OS (p=0.002, p=0.012, respectively).

As shown in Table 3, the prognostic impact of a high density of CD3^+^, CD8^+^ and FoxP3^+^ lymphocytes in relation to TTR, using the CRT based cutoff, was confirmed in univariable Cox regression analysis. In multivariable analysis, high CD8^+^ and FoxP3^+^ density remained independent factors of a prolonged TTR (HR=0.30, 95% CI=0.12-0.77, and HR=0.55, 95% CI=0.34-0.89, respectively). The prognostic impact of a high CD8^+^, FoxP3^+^ and NKp46^+^ density in relation to OS using the CRT based cutoff was also confirmed in univariable Cox regression analysis (Table 3). In multivariable analysis, only high CD8^+^ density remained an independent prognostic factor (HR=0.10, 95% CI=0.01-0.73).

**Table 3:** Cox proportional hazards analysis of the impact of investigative lymphocyte subsets on time to recurrence and overall survival in the entire cohort

	TTR			OS		
	n (events)	HR (95 % CI)	*P*	n (events)	HR (95 % CI)	*P*
**CD3^+^**						
*Univariable*						
Low	59	1.00		59	1.00	
High	87	0.58 (0.37-0.89)	0.014	113	0.72 (0.50-1.03)	0.075
*Multivariable*						
Low	59	1.00		59	1.00	
High	87	0.64 (0.40-1.04)	0.070	113	0.86 (0.57-1.28)	0.450
**CD8^+^**						
*Univariable*						
Low	123	1.00		165	1.00	
High	23	0.31 (0.12-0.75)	0.010	6	0.11 (0.02-0.81)	0.030
*Multivariable*						
Low	123	1.00		165	1.00	
High	23	0.30 (0.12-0.77)	0.013	6	0.10 (0.01-0.73)	0.023
**FoxP3^+^**						
*Univariable*						
Low	52	1.00		166	1.00	
High	95	0.44 (0.28-0.68)	<0.001	7	0.11 (0.02-0.80)	0.029
*Multivariable*						
Low	52	1.00		166	1.00	
High	95	0.55 (0.34-0.89)	0.015	7	0.24 (0.03-1.77)	0.162
**NKp46^+^**						
*Univariable*						
Low	60	1.00		138	1.00	
High	80	0.65 (0.41-1.01)	0.056	27	0.47 (0.27-0.83)	0.009
*Multivariable*						
Low	60	1.00		138	1.00	
High	80	1.35 (0.81-2.23)	0.246	27	0.69 (0.38-1.24)	0.213

Classification and regression tree-derived prognostic cutoffs are used to define “low” and “high” in all analyses. TTR= time to recurrence, OS= overall survival, HR= hazard ratio. Multivariable analysis includes age, location, adjuvant treatment, T-stage, N-stage, M-stage, differentiation grade (high vs low), resection margin (R0, R1, R2).

The associations of high and low lymphocyte density, defined by the median cutoff value, with OS in the entire cohort and according to tumor location are shown in Table 4. In the entire cohort the significant associations between high density of FoxP3^+^ and NKp46^+^ lymphocytes with a prolonged OS were confirmed in univariable analyses. In multivariable analysis, only high NKp46^+^ density remained an independent prognostic factor (HR=0.61, 95% CI=0.41-0.90). In esophageal tumors, high FoxP3^+^ density was significantly associated with a prolonged OS in univariable but not in multivariable Cox regression analysis. High density of NKp46^+^ cells was however significantly associated with a prolonged OS in both univariable and multivariable analysis (HR=0.49, 95% CI=0.28-0.86). In gastric tumors, only high FoxP3^+^ density was significantly associated with a prolonged OS in the univariable model, but this association did not remain significant in the multivariable analysis.

**Table 4:** Cox proportional hazards analysis of the impact of investigative lymphocyte subsets on overall survival in the entire cohort, esophageal and gastric cancer, respectively

	Entire cohort			Esophagus			Stomach		
	n (events)	HR (95 % CI)	*P*	n (events)	HR (95 % CI)	*P*	n (events)	HR (95 % CI)	*P*
**CD3^+^**									
*Univariable*									
Low	86	1.00		57	1.00		29	1.00	
High	86	0.87(0.61-1.24)	*0.439*	40	0.71 (0.43-1.15)	*0.165*	46	1.02 (0.60-1.74)	*0.951*
*Multivariable*									
Low	86	1.00		57	1.00		29	1.00	
High	86	1.04(0.68-1.58)	*0.855*	40	0.95(0.52-1.72)	*0.857*	46	1.13(0.56-2.29)	*0.729*
**CD8^+^**									
*Univariable*									
Low	86	1.00		57	1.00		29	1.00	
High	85	0.92(0.65-1.31)	*0.651*	39	0.79 (0.49-1.29)	*0.354*	46	1.01 (0.59-1.74)	*0.966*
*Multivariable*									
Low	86	1.00		57	1.00		29	1.00	
High	85	1.00(0.67-1.5	*0.999*	39	0.98(0.54-1.75)	*0.932*	46	0.94(0.49-1.81)	*0.858*
**FoxP3^+^**		0)							
*Univariable*									
Low	87	1.00		53	1.00		34	1.00	
High	86	0.58(0.41-0.83)	*0.003*	45	0.54(0.34-0.88)	*0.014*	41	0.58 (0.34-0.99)	*0.045*
*Multivariable*									
Low	87	1.00		53	1.00		34	1.00	
High	86	0.88(0.59-1.30)	*0.515*	45	0.96(0.54-1.72)	*0.895*	41	0.76(0.41-1.42)	*0.387*
**NKp46^+^**									
*Univariable*									
Low	93	1.00		57	1.00		36	1.00	
High	72	0.63(0.43-0.90)	*0.012*	37	0.50 (0.30-0.83)	*0.008*	35	0.78(0.46-1.35)	*0.379*
*Multivariable*									
Low	93	1.00		57	1.00		36	1.00	
High	72	0.61 (0.41-0.90)	*0.014*	37	0.49(0.28-0.86)	*0.012*	35	0.84(0.41-1.70)	*0.619*

Prognostic cutoffs derived from the median number of immune cells are used to define “low” and “high” in all analyses. OS= overall survival, HR= hazard ratio. CI= confidence interval. Multivariable analysis includes age, location (entire cohort), adjuvant treatment, T-stage, N-stage, M-stage, differentiation grade (high vs low), resection margin (R0, R1, R2). Low and high defined with median value as cutoff.

The prognostic value was not improved for any of the lymphocyte subsets when each compartment (intratumoral, tumor-adjacent or stromal) was analysed separately (data not shown).

### Prognostic significance of T, NK/T and NK cells in relation to B cell and plasma cell density

**Figure 4:**
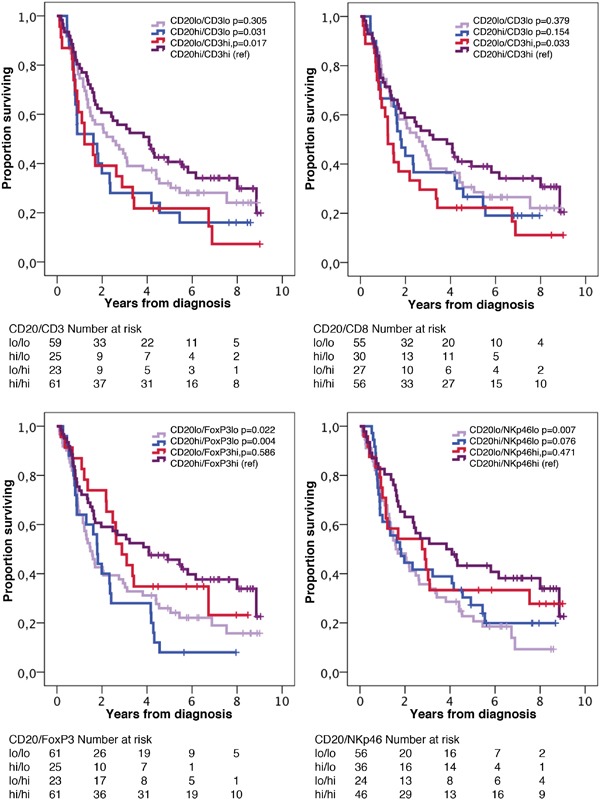
Kaplan-Meier estimates of overall survival in strata according to combinations of high and low density of CD20^+^ cells and CD3^+^, CD8^+^, FoxP3^+^ and NKp46^+^ cells, respectively, in the entire cohort The median value is used as cut off for all lymphocyte subsets.

**Figure 5:**
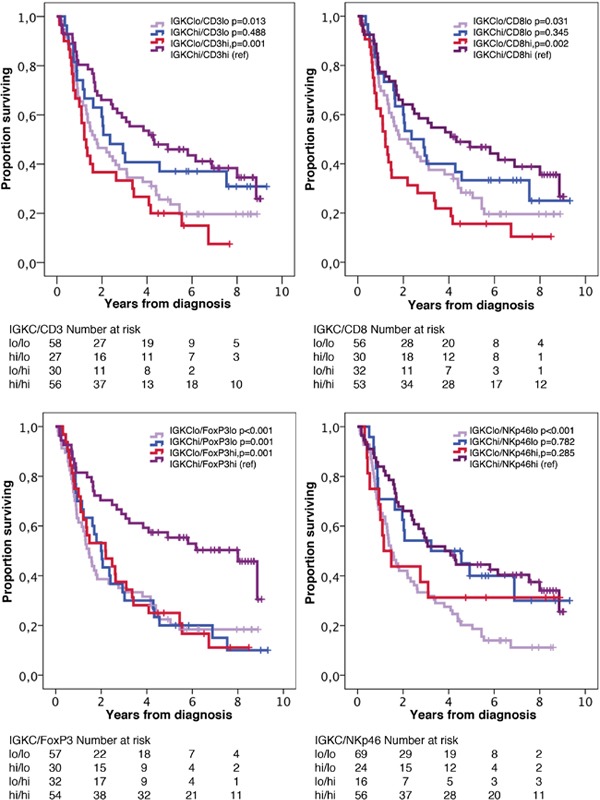
Kaplan-Meier estimates of overall survival in strata according to combinations of high and low density of IGKC^+^ cells and CD3^+^, CD8^+^, FoxP3^+^ and NKp46^+^ cells, respectively, in the entire cohort The median value is used as cut off for all lymphocyte subsets.

Next, we examined the impact of the investigated T and NK lymphocytes on OS in relation to B lymphocyte and plasma cell density, using the median value as cutoff. As shown in Figures 4 and 5, the best prognosis was observed for patients with tumors displaying high density of either CD20^+^ B cells or IGKC^+^ plasma cells combined with high density of either high CD3^+^, CD8^+^, FoxP3^+^ or NKp46^+^ lymphocytes.

As shown in Table 5, a significant interaction was observed for both CD3^+^ and CD8^+^ cells with CD20^+^ cells in relation to OS in the entire cohort (p_interaction_ =0.009 and 0.41, respectively). In esophageal tumors, there was only a significant interaction for CD3^+^ cells and CD20^+^ cells (p_interaction_ =0.028), whereas in gastric tumors, significant interactions were observed for CD3^+^, CD8^+^ and FoxP3^+^ cells with CD20^+^ cells in relation to OS (p_interaction_ =0.012, 0.009 and 0.007, respectively). As further shown in Table 5, a significant interaction was observed for FoxP3^+^ cells with IGKC^+^ cells in relation to OS in the entire cohort (p_interaction_ =0.023). In esophageal tumors, no significant interactions were observed for the investigated T lymphocytes or NK cells with IGKC^+^ cells, but in gastric tumors, a significant interaction was observed for FoxP3^+^ cells with IGKC^+^ cells (p_interaction_ =0.034). The analyses were also performed in M0 cases only (Table 6), yelding similar results.

**Table 5:** Cox proportional hazards analysis of overall survival in relation to high and low infiltration of T and NK cells stratified by B cell and plasma cell density

	CD20^+^					IGKC^+^				
	Low			High		Low			High	
	n	HR (95% CI)	n	HR (95% CI)	*p for interaction*	n	HR (95% CI)	n	HR (95% CI)	*p for interaction*
**Entire cohort**										
**CD3^+^**										
Low	59	1.00	25	1.00	*0.009*	58	1.00	27	1.00	*0.208*
High	23	1.54(0.90-2.62)	61	0.56(0.33-0.96)		30	1.29(0.80-2.09)	56	0.82(0.46-1.45)	
**CD8^+^**										
Low	55	1.00	30	1.00	*0.041*	56	1.00	30	1.00	*0.078*
High	27	1.48(0.89-2.48)	56	0.69(0.41-1.15)		32	1.49(0.93-2.40)	53	0.77(0.44-1.33)	
**FoxP3^+^**										
Low	61	1.00	25	1.00	*0.327*	57	1.00	30	1.00	*0.023*
High	23	0.69(0.39-1.22)	61	0.47(0.27-0.80)		32	0.97(0.60-1.56)	54	0.42(0.25-0.73)	
**NKp46^+^**										
Low	56	1.00	36	1.00	*0.821*	69	1.00	24	1.00	*0.575*
High	24	0.70(0.40-1.23)	46	0.62(0.37-1.06)		16	0.76(0.40-1.44)	56	0.92(0.50-1.68)	
**Esophageal cancer**										
**CD3^+^**										
Low	38	1.00	17	1.00	0.028	36	1.00	20	1.00	0.381
High	15	1.09 (0.55-2.16)	24	0.37(0.18-0.78)		16	0.96(0.50-1.85)	24	0.61(0.28-1.33)	
**CD8^+^**										
Low	35	1.00	21	1.00	0.207	36	1.00	21	1.00	0.402
High	18	1.06(0.55-2.04)	20	0.58(0.28-1.22)		16	1.10(0.57-2.12)	23	0.72(0.34-1.53)	
**FoxP3^+^**										
Low	38	1.00	14	1.00	0.818	33	1.00	20	1.00	0.112
High	17	0.49(0.24-1.00)	27	0.57(0.27-1.19)		20	0.84(0.45-1.57)	25	0.38(0.17-0.82)	
**NKp46^+^**										
Low	37	1.00	19	1.00	0.514	42	1.00	15	1.00	0.151
High	15	0.40(0.18-0.87)	21	0.60(0.29-1.27)		10	0.37(0.14-0.94)	27	0.93(0.41-2.10)	
**Gastric cancer**										
**CD3^+^**										
Low	21	1.00	8	1.00	*0.012*	22	1.00	7	1.00	*0.292*
High	8	5.26(1.86-14.91)	37	0.96(0.39-2.32)		14	1.94(0.93-4.06)	32	1.00(0.38-2.68)	
**CD8^+^**										
Low	20	1.00	9	1.00	*0.009*	20	1.00	9	1.00	*0.063*
High	9	4.36(1.63-11.63)	36	0.84(0.36-1.96)		16	2.12(1.01-4.45)	30	0.68(0.28-1.65)	
**FoxP3^+^**										
Low	23	1.00	11	1.00	*0.007*	24	1.00	10	1.00	*0.034*
High	6	1.90(0.73-4.91)	34	0.38(0.18-0.83)		12	1.24(0.59-2.61)	29	0.36(0.16-0.82)	
**NKp46^+^**										
Low	19	1.00	17	1.00	*0.066*	27	1.00	9	1.00	*0.109*
High	9	1.84(0.79-4.29)	25	0.66(0.31-1.38)		6	2.71(1.04-7.02)	29	0.84(0.33-2.11)	

Prognostic cutoffs derived from the median number of immune cells are used to define “low” and “high” in all analyses. HR= hazard ratio. CI= confidence interval.

**Table 6:** Cox proportional hazards analysis of overall survival in patients with M0 disease in relation to high and low infiltration of T and NK cells stratified by B cell and plasma cell density

	CD20^+^					IGKC^+^				
	Low			High		Low			High	
	n	HR (95% CI)	n	HR (95% CI)	*p for interaction*	n	HR (95% CI)	n	HR (95% CI)	*p for interaction*
**Entire cohort**										
**CD3^+^**										
Low	52	1.00	20	1.00	*0.006*	51	1.00	22	1.00	*0.315*
High	23	1.71(0.98-2.95)	55	0.54(0.30-0.98)		28	1.33(0.78-2.21)	52	0.90(0.47-1.42)	
**CD8^+^**										
Low	48	1.00	26	1.00	*0.017*	51	1.00	24	1.00	*0.198*
High	26	1.65(0.96-2.83)	49	0.62(0.35-)		28	1.46(0.88-2.43)	49	0.84(0.45-1.58)	
**FoxP3^+^**										
Low	53	1.00	19	1.00	*0.225*	50	1.00	23	1.00	*0.043*
High	23	0.76(0.43-1.36)	56	0.46(0.25-0.83)		30	0.99(0.60-1.64)	51	0.45(0.25-0.82)	
**NKp46^+^**										
Low	51	1.00	29	1.00	*0.873*	62	1.00	19	1.00	*0.338*
High	22	0.67(0.37-1.21)	43	0.70(0.39-1.24)		15	0.75(0.38-1.47)	52	1.13(0.55-2.31)	
**Esophageal cancer**										
**CD3^+^**										
Low	34	1.00	14	1.00	0.015	32	1.00	17	1.00	0.508
High	15	1.19(0.59-2.39)	24	0.35(0.16-0.77)		16	0.99(0.51-1.94)	24	0.69(0.30-1.57)	
**CD8^+^**										
Low	31	1.00	18	1.00	0.194	33	1.00	17	1.00	0.807
High	17	1.12(0.56-2.23)	20	0.59(0.27-1.28)		15	1.09(0.55-2.15)	23	0.87(0.38-1.99)	
**FoxP3^+^**										
Low	33	1.00	11	1.00	0.990	19	1.00	16	1.00	0.188
High	17	0.53(0.26-1.11)	27	0.56(0.25-1.26)		20	0.87(0.46-1.65)	25	0.43(0.19-0.98)	
**NKp46^+^**										
Low	34	1.00	17	1.00	0.525	39	1.00	13	1.00	0.087
High	14	0.36(0.16-0.84)	20	0.58(0.27-1.29)		9	0.31(0.11-0.88)	26	1.03(0.42-2.53)	
**Gastric cancer**										
**CD3^+^**										
Low	18	1.00	6	1.00	*0.020*	19	1.00	5	1.00	*0.461*
High	8	6.74 (2.14-21.21)	31	1.12(0.38-3.28)		12	2.20(0.98-4.93)	28	1.24(0.36-4.27)	
**CD8^+^**										
Low	17	1.00	8	1.00	*0.003*	18	1.00	7	1.00	*0.090*
High	9	5.42(1.86-15.81)	29	0.74(0.29-1.88)		13	2.12(1.01-4.45)	26	0.68(0.24-1.91)	
**FoxP3^+^**										
Low	20	1.00	8	1.00	*0.006*	21	1.00	7	1.00	*0.045*
High	6	2.19(0.82-5.88)	29	0.34(0.14-0.83)		10	1.26(0.56-2.86)	26	0.35(0.13-0.89)	
**NKp46^+^**										
Low	17	1.00	12	1.00	*0.206*	23	1.00	6	1.00	*0.293*
High	8	1.84(0.79-4.29)	23	0.87(0.36-2.91)		6	3.46(1.27-9.43)	26	1.23(0.36-4.24)	

Prognostic cutoffs derived from the median number of immune cells are used to define “low” and “high” in all analyses. HR= hazard ratio. CI= confidence interval.

## DISCUSSION

In this study, we show for the first time that the prognostic value of tumor-infiltrating T lymphocytes in resected esophageal and gastric adenocarcinoma differs by the density of infiltrating B lymphocytes and plasma cells. The prognostic value of B cells and plasma cells in cancer has only recently begun to receive attention, and the likely existence of a functional interplay between T cells and B cells is highly relevant [20, 34]. The use of the relative T and B/plasma cell content as a biomarker has not yet been investigated in many major solid tumors [35], the exception being ovarian cancer where Kroeger et al. demonstrated that CD8^+^ TILs carried the most evident prognostic benefit only in the presence of plasma cells and CD20^+^ TILs [25]. Concordant findings were found when cases with M1 disease were excluded, and subgroup analysis according to tumor location revealed that the majority of significant prognostic interactions between B cells and T cells were observed in gastric cancer. However, given the rather small number of cases in each category, these findings need to be validated in larger cohorts of esophageal and gastric cancer, respectively, before any conclusions can be drawn regarding potential biological differences underlying these observations.

In line with several previous studies, high density of CD3^+^, CD8^+^ and FoxP3^+^ T lymphocytes was in general significantly associated with a favorable prognosis, regarding either or both TTR and OS. Lee et al examined the density of CD3^+^, CD8^+^ and CD20^+^ TILs in resected tumors from 220 neoadjuvant chemoradiotherapy-naïve patients with gastric cancers, demonstrating that high infiltration of both CD3^+^ and CD8^+^ TILs were independent favorable prognostic factors respectively [10]. Moreover CD20^+^ density was not found to be prognostic in the study by Lee et al [10], which is also in line with a previous study using the present cohort [33]. In another study, high density of FoxP3^+^ stromal TILs was found to be an independent favorable prognostic factor, but no prognostic impact was demonstrated for CD3^+^, CD8^+^ or CD20^+^ TILs, in chemoradiotherapy-naïve tumors from 52 patients with radically resected, distant metastasis free, gastric cancer of the cardia [13]. However, contrasting findings have also been shown where infiltration of FoxP3^+^ TILs was found to be associated with poor prognosis in gastric cancer patients [12, 36].

The number of studies related to the prognostic significance of T-lymphocytes is more sparse with regards to esophageal cancer, but the density of CD8^+^ and FoxP3^+^ TILs in tumors from 196 patients, all of whom had received neoadjuvant chemoradiotherapy, was found to be associated with a reduced cancer-specific survival [15]. It is not clear whether that study included squamous cell carcinoma, adenocarcinoma, or both.

In this study we also examined the prognostic significance of NK cells as determined by NKp46 positivity. The results demonstrated that high density of NKp46^+^ cells, using the median value as cutoff, was an independent predictor of a prolonged OS in the entire cohort and in esophageal, but not in gastric cancer. We believe these results to be new, as we are not aware of any previous study on the occurrence and impact on clinical outcome of NK cells in gastric or esophageal tumors. However, indirect evidence of an association of NK cells with a favorable prognosis is provided in a study by Mimura et al., encompassing 102 patients with gastric cancer, where tumor-specific expression of the NKG2D ligands MICA/B and ULBP2 was found to be significantly associated with an improved survival [37].

As noted above, the findings regarding the prognostic impact of different subsets of T cells in esophageal and gastric cancer are ambiguous and more studies are necessary to clarify the role of the immune system in these neoplasms. Therefore, the results from the present study regarding the prognostic interaction between tumor-infiltrating B and plasma cells as well as T cells are of potential importance, since they implicate the existence of synergistic mechanisms that influence tumor progression and prognosis, and, consequently, the prognostic value of different T cell subsets or NK cells alone.

The use of the TMA-technique may pose a limitation to the study that needs to be acknowledged. Most TMAs, including the herein used, have been constructed to represent the tumoral rather than the stromal compartment, which may render them less suitable for analyses of the tumor microenvironment. It should however be pointed out that previous studies have demonstrated concordant results regarding the prognostic value of high infiltration of B cells in colorectal cancer, as demonstrated both by the use of whole tissue sections [38] and by the use of TMA [21], and the herein presented results regarding the prognostic value of CD3^+^, CD8^+^ and FoxP3^+^ TILs in gastric cancer [10, 13]. It can also be argued that analysis of one single whole tissue section may not be the ideal way of assessing the density of TILs in the tumor microenvironment, as it will still not accurately reflect a potential heterogeneity. In an attempt to reduce sampling bias, the TMA used in the present study was constructed with duplicate tissue cores from two different paraffin blocks with primary tumor, although the stromal compartment was not specifically sampled. The TMA technique may however be an ideal tool for studies on the prognostic and predictive value of TILs, as it enables a comprehensive sampling of tissue representing both tumor nest and stroma from multiple tissue blocks.

Another potential caveat is that when many statistical analyses are performed, there is a risk of type I statistical errors, i.e. detection of significances that are coincidental. This can be circumvented by the Bonferroni correction method, whereby the selected p-value threshold is divided by the number of tests made. This method has however disadvantages as it increases the risk for type II errors, i.e. that some significant associations are not detected. As the nature of this paper is exploratory rather than confirmatory, setting the significance levels too high may obscure som potentially important findings.

The herein examined tumors are derived from a clinically and histopathologically well characterized, consecutive cohort of patients with chemoradiotherapy-naïve tumors, of whom only a minor proportion had received adjuvant treatment. As chemotherapy is known to potentially alter the composition of the immune infiltrate, and thus possibly clouding the natural prognostic effects of the immune cells, this study population is well suitable for research on the potential prognostic impact of TILs [39]. However, given the observed prognostic interaction between T cells and B cells, future studies should also explore whether such an interaction exists in the predictive setting, and how neoadjuvant chemo- or chemoradiotherapy affects the density and prognostic effects of various subsets of TILs. In addition, the role of B cells in immune checkpoint inhibitor therapy has not yet been fully examined and may be of importance, since it has lately been shown that also T cells can be dependent on B cell activation [40].

In conclusion, the findings from the present study indicate, for the first time, a synergistic beneficial prognostic effect of high density of T cells and B cells in the tumor microenvironment of esophageal and gastric adenocarcinoma, in particular for the latter. These findings give important directions for future clinical as well as functional studies in all types of solid cancer, as they suggest that combined analysis of B and T cells might give a more detailed understanding of the prognostic and predictive role of the immune microenvironment, thus increasing the probability of their successful translation into clinical practice.

## MATERIALS AND METHODS

### Patients

### Study design and participants

This study is based on a cohort that has been described in several previous studies [26–32]. It encompasses a consecutive series of 174 patients with chemoradiotherapy-naïve esophageal and gastric adenocarcinoma, the latter including the esophagogastric junction. All patients were subjected to surgical resection at the University Hospitals of Lund and Malmö between January 1, 2006 and December 31, 2010. Tumor location was based on endoscopy findings and classification of the tumor stage was done according to the 7th edition of the UICC/AJCC TNM classification [41]. Clinical data regarding recurrence and vital status were obtained retrospectively from medical records and the last update, with additional re-examination of some of the clinicopathological data, reaches until March 2016. TTR was defined as time from diagnosis (date of result of the preoperative biopsy) to the date of proven recurrent disease (local, regional or distant) by biopsy or radiology. TTR was not calculated for patients with M1 disease or macroscopic residual tumor (R2). OS was defined as time from diagnosis (date of result of the preoperative biopsy) to the date of death from any cause. Residual tumor status was classified as: R0 = no residual tumor (free resection margins according to pathology report), R1 = microscopic residual tumor (narrow or compromised resection margins according to pathology report), R2 = macroscopic residual tumor (according to surgery report). Three patients in the cohort had known M1-disease (distant metastases) at the time of surgery and therefore underwent surgery with a palliative intent, with the aim to decrease symptoms from the primary tumor. The rest of the cohort, 98.7%, was operated on with a curative intent, but in 16 of these patients, M1-disease was detected either perioperatively or postoperatively through pathological examination of the resected specimen. Neoadjuvant or perioperative oncological therapy was not given to any of the patients in this cohort. Postoperative adjuvant treatment was given to 7.5% (n = 13), of which 11 patients were given chemoradiotherapy, 1 patient chemotherapy, and 1 patient radiotherapy.

### Ethical considerations

All EU and national regulations and requirements for handling human samples have been fully complied with during the conduct of this project; i.e. decision no. 1110/94/EC of the European Parliament and of the Council (OJL126 18,5,94), the Helsinki Declaration on ethical principles for medical research involving human subjects, and the EU Council Convention on human rights and Biomedicine. The study was approved by the regional ethics committee at Lund University (No. 445/07), whereby the committee waived no need for consent other than by the option to opt out.

### Tissue microarray construction and immunohistochemistry

Tissue microarrays (TMAs) were constructed as previously described using a semiautomated arraying device (TMArrayer, Pathology Devices, Westminister, MD, USA) [26]. Duplicate tissue cores (1 mm) were obtained from two different blocks of the primary tumor.

For immunohistochemical analysis of FoxP3 and CD8, 4 μm TMA-sections were automatically pre-treated using the PT Link system and then stained in an Autostainer Plus (Dako; Glostrup, Denmark) with the anti-FoxP3 antibody (clone 236A/E7, mouse, dilution 1:200, Abcam, Cambridge, UK), and the anti-CD8 antibody (clone C8/144B, mouse; dilution, 1:50; product M7103; Dako). For immunohistochemical analysis of CD3, 4 μm TMA-sections were pretreated using ULTRA Cell Conditioning Solution 1, pH 8.5 (Ventana Medical Systems Inc.Tucson, AZ, USA) for heat induced epitope retrieval, and stained in a Ventana BenchMark stainer (Ventana Medical Systems Inc.) with the anti-CD3 antibody (clone 2GV6, pre-diluted, Ventana Medical Systems Inc).

Analysis of CD20 and IGKC was performed as previously described [33].

### Evaluation of the immunohistochemical staining and lymphocyte count

The total number (intratumoral, tumor-adjacent and stromal) CD3^+^ and CD8^+^ immune cells in each core was calculated by automated analysis using the co-localization algorithm within the Halo image analysis software (Indica Labs, Corrales, NM, USA). The number of FoxP3^+^ and NKp46^+^ cells (intratumoral, tumor-adjacent and stromal) was calculated manually by two independent investigators (MCS and KJ), the latter being a board certified pathologist. Discrepant cases were re-evaluated again and discussed in order to reach consensus. A mean value of the two cores was calculated and used in the analyses.

### Statistics

Mann-Whitney U and Kruskal Wallis tests were used to evaluate associations between the counted immune cells and established clinicopathological factors and to detect differences in distributions between morphological subtypes. Spearman's rank correlation test was used to detect associations between subsets of immune cells. Classification and regression tree (CRT) analysis was used to determine prognostic cut-off points for the survival analyses. Dichotomized variables based on the median values were also constructed. Kaplan-Meier analysis and the log rank test were applied to detect differences in time to recurrence (TTR) and overall survival (OS). Cox proportional hazards models were used to calculate univariable and multivariable hazard ratios for TTR and OS. The multivariable model included age, location (entire cohort), adjuvant treatment, T stage, N stage, M stage, differentiation grade, and residual tumor status. For analysis of the interaction between adjuvant treatment and high and low density of the different lymphocyte subsets, an interaction variable was constructed of adjuvant treatment (+/−) × lymphocyte density (high/low). All tests were two-tailed and p-values of 0.05 or less were considered significant. All calculations were performed using IBM SPSS Statistics for Mac version 24.0 (IBM, Armonk, NY, USA).

## Abbreviations

NK cell: natural killer cell
TILs: tumor-infiltrating lymphocytes
Tregs: regulatory T cells
CRT: classification and regression tree
HR: hazard ratio
IHC: immunohistochemistry
IGKC: immunoglobulin kappa C
OS: overall survival
TMA: tissue microarray
TTR: time to recurrence
